# Adherence to Nucleoside/Nucleotide Analogue Treatment in Patients with Chronic Hepatitis B

**DOI:** 10.4274/balkanmedj.2016.1461

**Published:** 2017-12-01

**Authors:** Emin Ediz Tütüncü, Rahmet Güner, Yunus Gürbüz, Ayşe Kaya Kalem, Barış Öztürk, İmran Hasanoğlu, İrfan Şencan, Mehmet A. Taşyaran

**Affiliations:** 1 Department of Infectious Diseases and Clinical Microbiology, University of Health Sciences, Dışkapı Yıldırım Beyazıt Training and Research Hospital, Ankara, Turkey; 2 Department of Infectious Diseases and Clinical Microbiology, Ankara Yıldırım Beyazıt University School of Medicine, Ankara, Turkey; 3 Clinic of Infectious Diseases and Clinical Microbiology, Ankara Atatürk Training and Research Hospital, Ankara, Turkey

**Keywords:** Medication adherence, chronic hepatitis B, treatment outcome, nucleoside/nucleotide analogues

## Abstract

**Background:**

Adherence to medication is an important aspect of preventing drug resistance and treatment failure in patients receiving nucleos(t)ide analogues for chronic hepatitis B.

**Aims:**

To assess adherence to nucleoside/nucleotide analogues in chronic hepatitis B treatment and to determine factors associated with non-adherence.

**Study Design:**

Cross-sectional study.

**Methods:**

The study enrolled 85 chronic hepatitis B patients who had been receiving nucleoside/nucleotide analogues for ≥3 months. A questionnaire was completed by patients themselves, and adherence was evaluated based on patients’ self-reporting. The use of at least 95% of the drugs in the previous month was considered as adequate adherence.

**Results:**

Adherence was adequate in 82.4% of patients. Female gender (p=0.003), unemployment (p=0.041) and lower monthly family income (p=0.001) were related to lower adherence. Better adherence was significantly linked to adequate basic knowledge regarding chronic hepatitis B (p=0.049), longer treatment duration than 12 months (p<0.001), previous use of other medications for chronic hepatitis B (p=0.014) and regular follow-up by the same physician (p<0.001).

**Conclusion:**

Counselling patients about their disease state and the consequences of non-adherence is an important intervention for enhancing adherence. Naïve patients should be followed up more frequently to reinforce adherence.

The current treatment guidelines recommend the use of nucleoside/nucleotide analogues (NAs) for the treatment of chronic hepatitis B (CHB). These drugs effectively suppress hepatitis B virus replication and are usually administered until specific endpoints are achieved. The endpoint for stopping treatment is unclear, particularly for hepatitis B e-antigen-negative patients, and the duration of treatment is unpredictable (1,2). Therefore, most patients with CHB require an extended duration of therapy, possibly for life. Though NAs are generally well tolerated with minimal side effects, long-term treatment can cause substantial problems such as antiviral resistance, nonadherence and the requirement for more expensive combination therapies (3).

Adherence to antiviral therapy is crucial for achieving sustained virological suppression and prevention of drug resistance. It was clearly shown that poor adherence to therapy was the main reason for treatment failure in patients treated with entecavir who previously achieved complete viral suppression. Non-adherence was also found to be a significant cause of virologic breakthrough in patients receiving adefovir (3). Discontinuation of medications can put patients at serious risk for hepatic flares and even decompensation.

Adherence rates are usually lower among patients with chronic conditions as compared with those with acute conditions (4). Indefinite duration of CHB therapy may be a risk factor for non-adherence to treatment. Medication adherence research has found patient non-adherence in a variety of conditions such as diabetes, hypertension, pulmonary diseases and HIV infection (5). Even though adherence to antiretroviral treatment has been widely studied in the literature, there are only a few reports regarding adherence to CHB treatment (3,6). The aims of this study were to determine the adherence rates to NA therapy in patients with CHB and the factors associated with adherence to treatment.

## MATERIALS AND METHODS

This cross-sectional survey study was conducted in out-patient clinics of Dışkapı Yıldırım Beyazıt Training and Research Hospital and Ankara Atatürk Training and Research Hospital, Ankara, Turkey. Consecutive CHB patients receiving NAs for more than 3 months were enrolled in the study. The purpose of the study was explained to the patients during routine visits, and those patients who volunteered to participate and gave informed consent were included. This study was approved by the institutional research ethics committee of Dışkapı Yıldırım Beyazıt Training and Research Hospital.

The patients completed a self-administered questionnaire. Socio-demographic data (age, sex, educational and employment status, monthly family income), history of hepatitis B virus infection, previous and current treatments, treatment modifications during follow-up and the presence of side effects were recorded.

Adherence to CHB therapy was evaluated subjectively based on patients’ self-reporting. Patients were asked about the number of doses missed within the previous week and month and the reasons for missing doses. The use of at least 95% of the drugs (i.e. missing medications ≤1 day in the previous month) was considered as adequate adherence.

Patients’ basic knowledge about CHB was assessed with four statements: “CHB affects the liver”, “CHB is a lifelong disease”, “CHB can lead to cirrhosis and liver cancer” and “CHB can be treated using medications”. All answers were recorded as “yes”, “no” or “don’t know”. Patients who responded correctly to at least three statements were considered as having “adequate basic knowledge” about CHB. In addition, the following questions were used to assess patients’ level of information regarding drug therapy. “Do you know why you have to use this medication?”, “Do you know how long this medication will be used?” and “Are you informed about the side effects of this medication?”. Questions in the questionnaire were open-ended, and the attending physicians interpreted the answers to these questions. The patients who responded correctly to all three questions were considered as “well informed”. Patients’ awareness about the effectiveness of their current treatment and regular follow-up by the same physician were also recorded.

### Statistical analysis

Descriptive statistics were calculated for all study variables. Percentages were reported for categorical variables, and means ± standard deviation (SD) or medians (minimum-maximum) were reported for continuous variables. Patients were categorized according to adherence level, and all descriptive statistics were calculated for adherent and non-adherent patients. Differences between two groups were determined by the use of a chi-square test. A p-value of <0.05 was accepted as statistically significant. Statistical analyses were performed using SPSS software, version 15.0 (SPSS Inc., Chicago, IL, USA). Factors with a significant p-value in univariate analysis were included in a logistic model as independent predictors of non-adherence.

## RESULTS

A total of 85 patients were enrolled in the study. The median age was 46 (24-75) years. Sixty-one patients were male (71.8%) and 24 were female (28.2%). The mean time since diagnosis was 9.2 (SD 6.2, range 1-30) years, and the mean time on NA therapy was 22.8 (SD 17.4, range 3-108) months.

Of the 85 patients in the study group, 38 (44.7%) were naïve at the beginning of their current therapy and the remaining 47 (55.3%) were previously treated with either interferons and/or NAs. Regarding current medications taken by the study group, the most frequently used NA was entecavir (n=29). Twenty-four patients were using tenofovir, 17 patients were on lamivudine and 2 patients were using adefovir. The remaining 13 patients were using combination therapies (8 patients were using lamivudine and adefovir, 4 patients were using lamivudine and tenofovir and 1 patient was using entecavir and tenofovir).

Ten (11.8%) and 24 (28.2%) patients reported missing at least one dose of their medication in the last week and month, respectively. Fifteen patients (17.6%) reported missing their medications on more than one day in the previous month and were considered non-adherent to therapy. The most commonly stated reasons for missing a medication dose were forgetfulness, unavailability of the drug and being away from home.

Among the study group, 67 patients (78.8%) were classified as having adequate basic knowledge regarding CHB, and 65 patients (76.5%) were considered as well informed concerning drug therapy. Eight patients (9.4%) reported having side effects, most commonly fatigue (n=3) and headache (n=2). Thirty-one patients (36.5%) were high school or university graduates, and almost half of all patients (n=42) were unemployed. All patients were eligible for health insurance coverage provided under the Turkish social security system. Eleven patients refused to answer questions about monthly family income and were therefore excluded from the statistical analysis on the effect of income on adherence. Of the 74 patients who responded, 37 reported a monthly family income of <1000 Turkish liras (approximately 330 USD).

Socio-demographic and clinical variables were compared between adherent and non-adherent patients. Male gender (p=0.003), employment (p=0.041), higher family income (p=0.001), adequate basic knowledge regarding CHB (p=0.049), previous use of other medications for CHB (p=0.014), being in treatment for more than 12 months (p<0.001), and regular follow-up by the same physician (p<0.001) were associated with higher levels of adherence (Table 1). In multivariate analysis and after controlling for other factors, it was shown that regular follow-up by the same physician was associated with a 6.7-fold increase in adherence (OR_adj_: 6.7, 95% CI: 1.5-22.7), and being in treatment for more than 12 months was associated with a 6.6-fold increase in adherence (OR_adj_: 6.3, 95% CI: 1.5-27.6).

## DISCUSSION

Treatment adherence rates for chronic conditions vary greatly in clinical practice and are typically around 50% (7,8). Although one can assume that perfect adherence to a medication regimen means taking all drugs as prescribed, there is no consensus standard for what constitutes adequate adherence (4). Research has shown that less than 90% antiretroviral adherence was strongly associated with viral rebound and clinically significant resistance (9), and more than 95% adherence to antiretroviral treatment was required for the most favourable treatment outcomes (10). On the other hand, the level of adherence required to obtain optimal long-term benefit in the setting of CHB treatment has yet to be determined.

Adequate adherence was subjectively defined as an adherence rate of more than 95% in this study, and 82.4% of patients were found to be adherent to their medications. Chotiyaputta et al. (6) recently reported that 55.3% of patients had more than 90% adherence to NA treatment for CHB, which was considerably lower than our result. Adherence to antiretroviral treatment varies between 37% and 83% (5). A meta-analysis of North American studies indicated a pooled estimate of 55% of patients achieving adequate levels of adherence (11). The lower adherence rates to antiretroviral medication can be explained by the greater complexity of the treatment regimen and more frequent side effects (6).

The majority of published adherence studies indicate that age is related to adherence (12). A higher adherence rate among older patients had been observed in antiretroviral and NA treatments (6,10,13). This may be related to younger patients’ lower levels of concern regarding chronic diseases, unawareness of the importance of adherence and unfamiliarity with medication usage. Moreover, younger patients are likely to have other priorities in their daily life, and they may not be able to attend to treatment or spare the time waiting for clinic appointments (12). We did not find an association between age and adherence; younger and older patients were similarly adherent to therapy (80.6 vs. 83.3%).

Gender was associated with medication adherence in our study. We have shown that men were significantly more adherent to treatment than women (90.2 vs. 62.5%, p=0.003). This finding is also supported by a recent review that female gender often predicts lower adherence in HIV treatment (14).

Socioeconomic status has traditionally been defined by education, income and occupation (15). Education shapes future occupational opportunities and earning potential and provides knowledge to access health resources and information on disease and treatment (15,16). Poor living conditions resulting from low income could play a role in preventing patients from complying with treatment (16). Additionally, unemployment has been linked to lower levels of health status and leads to lower family income. Factors such as poverty, illiteracy, low level of education and unemployment have been reported to have a significant effect on adherence (15). Contrarily, Falagas et al. (16) reported that, although many available studies suggested a positive trend, socioeconomic status was not consistently associated with adherence to treatment among HIV-infected patients. Even though all patients had health insurance coverage in this study, unemployment and lower monthly family income were found to be significantly associated with non-adherence (p=0.041 and 0.001, respectively). In addition, although the difference was not statistically significant, high school or university graduates were more likely to adhere to their treatments (87.1 vs. 79.6%). Indeed, low socioeconomic status may put patients in the position of having to choose between competing priorities, such as demands to direct the limited resources available to meet the needs of the family, and may affect adherence to therapy (5). The cost of treatment is an important barrier to adherence for patients who must pay for their medications. We have shown that, even in the absence of cost of medications, lower socioeconomic status was associated with non-adherence to therapy.

Patients who were considered as having adequate basic knowledge about CHB were significantly more adherent to treatment (p=0.049). Increased understanding of the disease, awareness of long-term consequences and knowledge regarding availability of treatment options may improve adherence (17). Moreover, knowledge about disease complications (e.g. CHB can lead to cirrhosis and liver cancer) may alter patients’ views of the necessity and expected benefits of the treatment and consequently, enhance adherence to treatment.

Another finding of this study was that those patients who were considered as well informed about their medications were more likely to adhere to their treatment; however, the difference was not significant (84.6 vs. 75%, p=0.324). Patients must understand what they are supposed to do before they can follow recommendations (18). Proper communication between the physician and the patient and clarification of the purpose and benefits of treatment are important factors affecting medication adherence (7,18,19). In this regard, it should be remembered that therapeutic education of patients in clinical practice effectively improves adherence to treatment in chronic diseases (4,20). Before starting a medication, all patients should be informed precisely regarding their disease state, the purpose of the therapy, the use of medications and the consequences of non-adherence. Furthermore, patients’ counselling should not be limited to the time of the initiation of treatment but should be continuous throughout the follow-up period and the importance of adherence should be addressed at each medical visit.

It was concluded that the patient-physician relationship is a strong factor that affects patients’ adherence (12). In large medical centres, patients may be seen by different physicians each time. We have shown in this study that attending regular follow-up appointments by the same physician correlates with better medication adherence (p<0.001), and it was revealed as an independent predictor of adherence in the logistic regression model (OR_adj_: 6.7, 95% CI: 1.5-22.7). This may be due to continued counselling (17), and familiarity with the doctor may make patients feel more comfortable to express concerns and ask questions about their health and treatment outcomes. Additionally, continuity of care and familiarity may improve physicians’ proficiency with a given patient (21). As a result, the physician-patient relationship should be individualized to improve adherence.

When patients start using a medication, they form beliefs about the necessity of the drug and formulate concerns about the consequences of its use. They will want to know whether it is working (22). It can be assumed that, if patients are aware that the current medication is successful, then they will be more motivated to use it. Hence, physicians should inform patients about the results of the follow-up tests and treatment outcomes. Indeed, we have noted a trend toward better adherence among patients who were aware of the effectiveness of their treatment with NAs.

Previously untreated patients and shorter duration of treatment were significantly associated with lower adherence (p=0.014 and lt;0.001, respectively) and being in treatment for more than 12 months was identified as an independent predictor of adherence (OR_adj_: 6.3, 95% CI: 1.5-27.6). Patients who start a new medication for a chronic condition experience considerable problems. The incidence of non-adherence was found to be greater with new than with existing medication (6,22). Although guidelines for the management of CHB recommend monitoring patients every 3-6 months, patients should be followed up more frequently during the first year of the treatment to reinforce adherence. One possible explanation for the superior adherence of treatment-experienced patients might be that a previous change in the treatment regimen (i.e. adding or switching to another agent) may change patients’ perspectives about the severity of the disease and the importance of adherence. A positive association between perceived disease severity threat and adherence has been shown (23). This was supported by another finding in this study that all patients who were using more than one drug were fully adherent to the treatment.

Although it is believed that the complexity of treatment threatens patients’ adherence, and the rate of adherence decreases as the number of daily doses increase (12), we have found no impact of the daily pill count on adherence. Once-daily dosing frequency of NAs helps to maximize adherence, and even those patients who require add-on regimens seem not to be affected by the number of daily doses.

In practice, side effects of the medication appear to be an important reason for non-adherence (12). Commonly used NAs have quite a few side effects compared with antiretroviral medications. Treatment discontinuation due to side effects was reported to be rare in patients receiving tenofovir and entecavir (24,25). We found no differences regarding the presence of side effects between adherent and non-adherent patients in this study. Thus, side effects of NAs do not seem to have an unfavourable impact on adherence to CHB treatment.

The main drawback of this study was that the adherence was evaluated on the basis of patients’ self-reporting. Although self-reported adherence is the most convenient method for evaluating adherence to a medication, it is subject to overestimation (4,9,13). In addition, due to the small sample size of this study, these findings are not generalizable. Nevertheless, these results could help to identify patients who tend to be non-adherent and highlight areas for improving adherence.

Treatment failure in patients receiving NAs may result from non-adherence, especially for potent drugs such as entecavir and tenofovir. Therefore, adherence should be ascertained in CHB patients who have a partial virologic response or virologic breakthrough while receiving NA treatment. In this regard, it should be borne in mind that adherence to medication should be questioned and reinforced at each visit, and efforts to improve adherence should be continued for as long as the medication is used. In addition, naïve patients should be followed up frequently during the first year of treatment to enhance adherence to NAs. Further larger studies will be required to determine the factors leading to non-adherence to NAs in order to improve adherence and treatment success in CHB.

## Figures and Tables

**Table 1:**
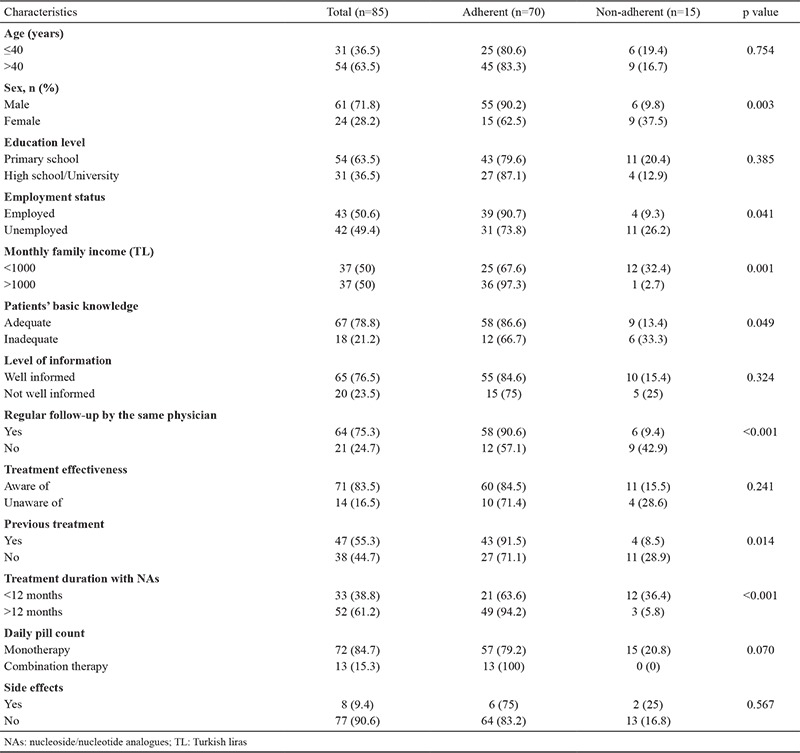
Socio-demographic and clinical variables according to adherence to treatment
